# Supplementary material for: Glycosylphosphatidylinositol-anchored proteins as chaperones and co-receptors for FERONIA receptor kinase signaling in Arabidopsis
Source: eLife. 2015 Jun 8;4:e06587. doi: 10.7554/eLife.06587 (PMC4458842; doi:10.7554/eLife.06587)
Supplement: Supplementary file 1. — A list of gene constructs. DOI: http://dx.doi.org/10.7554/eLife.06587.029 [file elife06587s001.docx]

**Supplemental Table 1. A list of gene constructs**

| **Constructs** | | **Description** | | **Reference** | |
| --- | --- | --- | --- | --- | --- |
| *LLG1* | | Genomic LLG1 fragment | | This work | |
| *pLLG1::GUS* | | LLG1 promoter::GUS | | This work | |
| *pLLG1::LLG1* | | LLG1 promoter::LLG1 cDNA | | This work | |
| *pLLG1::HA-LLG1** | | LLG1 promoter::HA-tagged LLG1 cDNA | | This work | |
| *35S::LLG1* | | CaMV 35S promoter::LLG1 cDNA | | This work | |
| *35S::GFP-LLG1** | | CaMV 35S promoter::GFP-LLG1 | | This work | |
| *35S::LLG1ΔC* | | CaMV 35S promoter::LLG1 with amino acid residues 148-168 deleted, just beyond the GPI-anchor attachment site | | This work | |
| MBP-LLG1 | | *E.coli* expressed maltose binding protein-tagged LLG1 | | This work | |
| MBP-LRE | | *E.coli* expressed maltose binding protein-tagged LRE | | This work | |
| MBP-ROP2 | | *E.coli* expressed maltose binding protein-tagged ROP2 | | (Duan et al., 2010) | |
| MBP-exJM | | *E. coli* expressed FER extracellular juxtamembrane (exJM) region (amino acid residues 385-447) | | This work | |
| His_6_-LLG1  His_6_-RALF1  MBP-RALF1 | | *E.coli* expressed His_6_-tagged LLG1  *E.coli* expressed His_6_-tagged mature RALF1  *E.coli* expressed maltose binding protein tagged mature RALF1 | | This work  This work  This work | |
| *pFER::FER-GFP** | | FER promoter::GFP-tagged FER | | (Duan et al., 2010) | |
| *35S::FER-GFP* | | CaMV 35S promoter::GFP-tagged FER | | This work | |
| *35S::FER-HA** | | CaMV 35S promoter::HA-tagged FER | | (Duan et al., 2010) | |
| *35S:: RFP-ER* | | CaMV 35S promoter::RFP-HDEL as an ER marker | | (Sinclair et al., 2009) | |
| *35S::VN-FER** &  *35S::YC-FER** | | CaMV 35S promoter::N-terminal Venus half-tagged FER & C-terminal YFP half-tagged FER | | This work | |
| *35S::VN-FERΔK**  &  *35S::YC-FERΔK** | | CaMV 35S promoter::N-terminal Venus half-tagged kinase domain-deleted FER & C-terminal YFP half-tagged kinase domain-deleted FER | | This work | |
| 35S::VN-LLG1* &  35S::YC-LLG1* | | CaMV 35S promoter::N-terminal Venus half-tagged LLG1 & C-terminal YFP half-tagged LLG1 | | This work | |
| 35S::AtARF1(Q71L) | | CaMV 35S promoter::AtARF1(Q71L) conversion to  constitutively GTP-bound form of the small GTPase | | (see Cai et al., 2011) | |
| *pFER::* and  *35S::FERΔexJM-GFP** | FER or 35S promoter expressed GFP-tagged FER deleted of the N-terminal juxtamembrane region amino acid residues 385-447 | | This work | |  |
| *AD-LLG1/LRE* and  *BD-LLG1/LRE*/ | Yeast-two-hybrid constructs with activation domain or  DNA binding domain fused to LLG1 or LRE | | This work | |  |
| *AD-exJM and*  *BD-exJM* | Yeast-two-hybrid constructs with activation domain or  DNA binding domain fused to FERexJM (amino acid residues 385-447) | | This work | |  |
| *35S::RbohD(N)-HA* | CaMV 35S promoter expressed N-terminal fragment of  *Arabidospsi* RbohD (amino acids 1 to 376) | | (see Wong et al., 2007) | |  |

*****construct shown in Figure 8.
